# Primary seminal vesicle Burkitt lymphoma in a patient living with HIV undergoing radical prostate and seminal vesicle resection: a rare missed case report

**DOI:** 10.1186/s13027-023-00509-8

**Published:** 2023-05-24

**Authors:** Menghua Wu, Xin Zheng, Wei Wang, Jing Chang, Meng Xue, Yu Zhang, Jian Song, Jimao Zhao

**Affiliations:** 1 Department of Urology, Beijing Youan Hospital, Capital Medical University, Beijing, China; 2 Department of Urology, Beijing Friendship Hospital, Capital Medical University, Beijing, China; 3 Department of Radiology, Beijing Youan Hospital, Capital Medical University, Beijing, China; 4 Department of Clinical Pathology, Beijing Youan Hospital, Capital Medical University, Beijing, China; 5 Department of Medical Record, Beijing Youan Hospital, Capital Medical University, Beijing, China

**Keywords:** PLWH, Burkitt lymphoma, Seminal vesicle, Missed case report

## Abstract

Primary seminal vesicle Burkitt lymphoma (PSBL) is rare that is not frequently reported. Burkitt lymphoma is often associated with extranodal organs. The diagnosis of carcinoma in seminal vesical can be difficult. In this report, we present a missed case of PSBL in a male patient who underwent radical prostate and seminal vesicle resection. We retrospectively analyzed the clinical data to explore the diagnosis, pathological features, treatment, and prognosis of this rare disease. The patient visited our hospital for dysuria, and the serum prostate-specific antigen (PSA) was moderately elevated. Pelvic magnetic resonance imaging (MRI) and computed tomography (CT) scans suggested a notable enlargement of the seminal vesicle. The patient then underwent radical surgery and the pathology diagnosis revealed Burkitt lymphoma. The diagnosis of PSBL is difficult, and the prognosis is generally poorer than that of other types of lymphoma. However, earlier diagnosis and treatment may help to improve the survival rate among patients with Burkitt lymphoma.

## Background

Burkitt lymphoma (BL) is a rare and aggressive type of non-Hodgkin lymphoma (NHL) that accounts for 1–2% of all NHL cases. Additionally, up to 40% of lymphomas in people living with HIV (PLWH) are BL [1, 2], who face a 10–20% lifetime risk of developing BL. Despite the introduction of antiretroviral therapy (ART), the incidence of BL in PLWH has not declined, unlike other NHLs associated with HIV, such as diffuse large B-cell lymphoma [1, 3]. The first clinical manifestation of HIV-associated BL is often extranodal site involvement, with gastrointestinal tract and the bone marrow involvement being the most common [4, 5]. In this case report, we describe a rare missed case of primary seminal vesicle Burkitt lymphoma (PSBL) in a PLWH.

## Case presentation

**Fig. 1:**
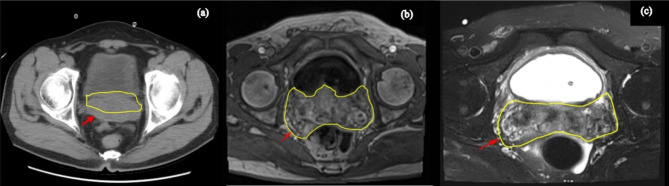
(**a**) Pelvic CT scan shows an obvious enlargement of the seminal vesical (**b**) Pelvic MRI T1 and (**c**) Pelvic MRI T2 suggest that the arrows point to a region of heterogeneous signal in the seminal vesicle

**Fig. 2:**
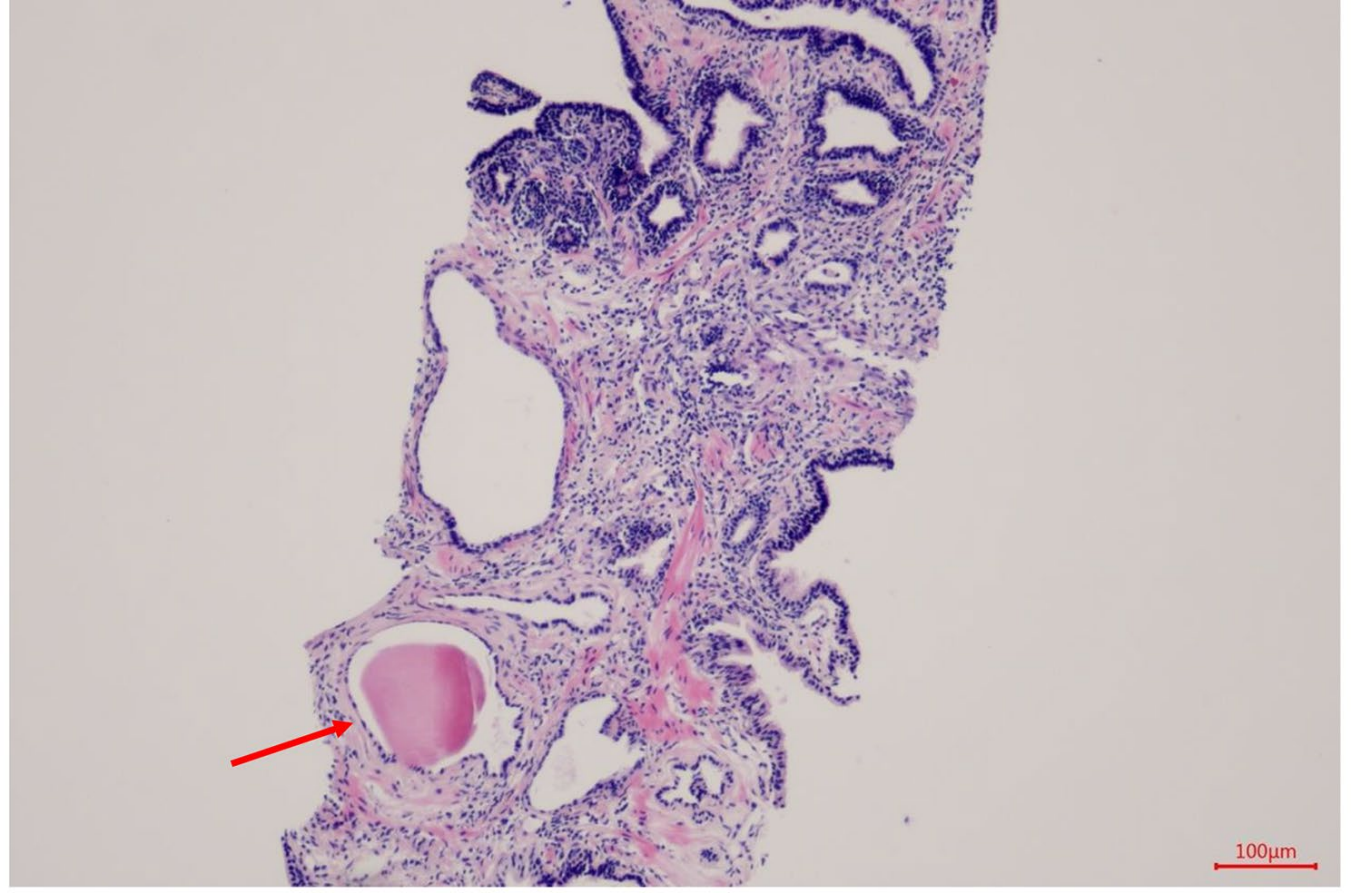
HE staining (100-fold) panel shows a glandular structure present, partial dilatation of the lumen, luminal secretions seen, interstitial hyperplasia, and no neoplastic lesions seen. the pathology shows benign prostate hyperplasia and chronic prostatitis

**Fig. 3:**
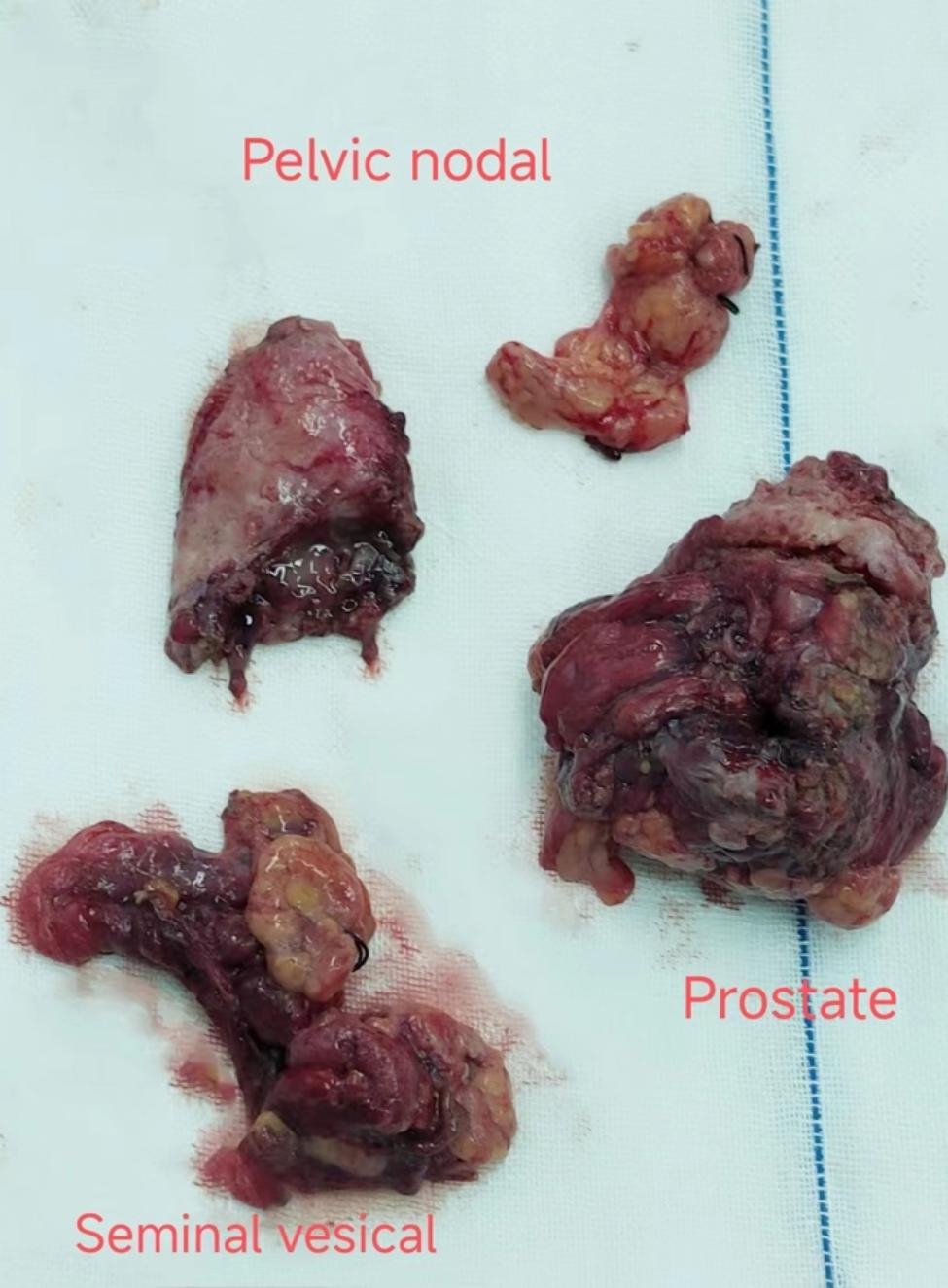
Pathological specimens after laparoscopic radical prostate and seminal vesical resection

**Fig. 4:**
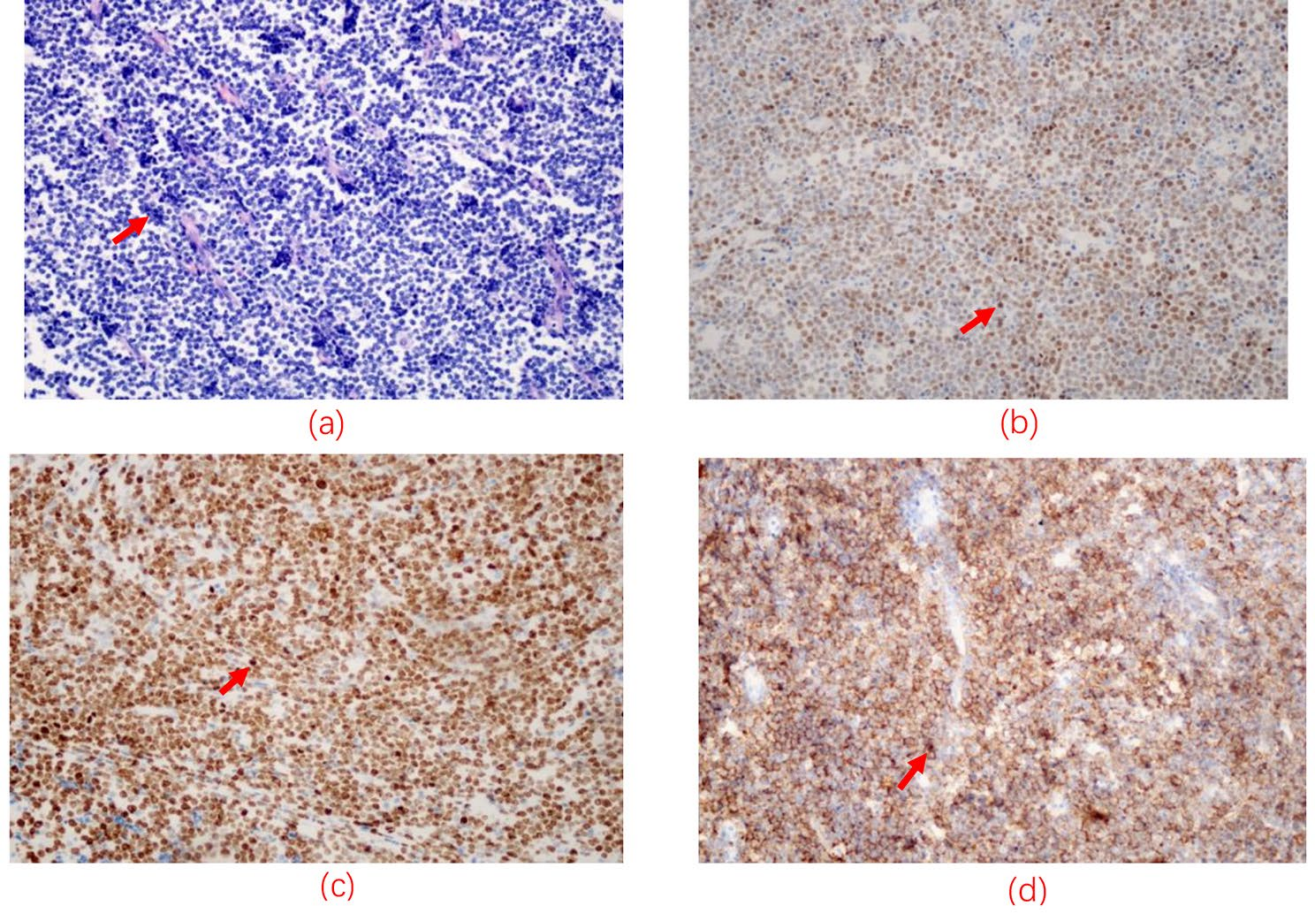
(**a**) Tumor cells were diffusely distributed, medium size, uniform in volume, with little cytoplasm, deep-stained nuclei, and homogeneous nuclear chromatin; immunohistochemistry; (**b**) Tumor cells were approximately 80% positive for c-myc (brownish granules); (**c**) Tumor cells were actively proliferating with > 90% positive for Ki-67(brownish granules); (**d**) Tumor cells were diffusely positive for the B-cell marker CD20(brownish granules)

**Fig. 5:**
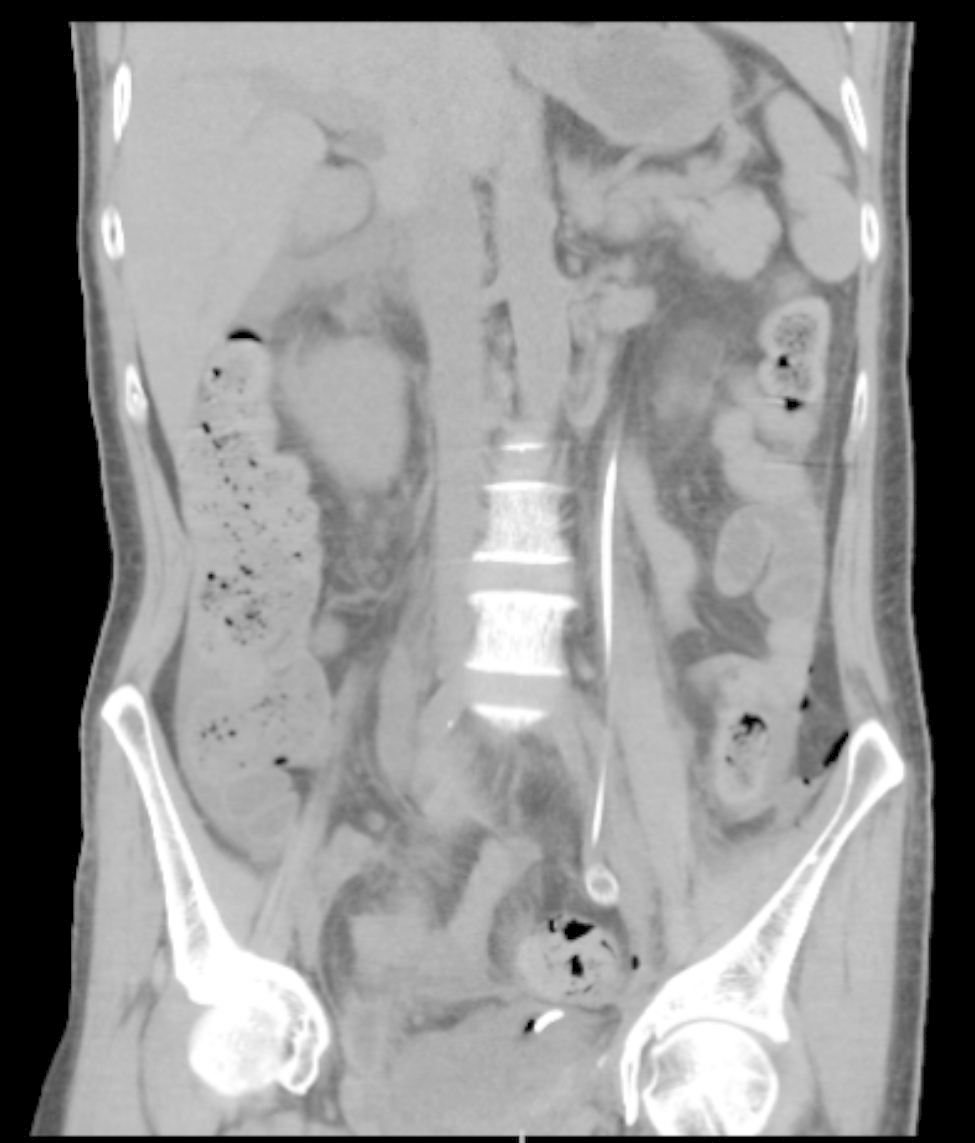
CT reconstruction suggests that the tumor progressed and induced bilateral hydronephrosis, only the left ureteral stent can be placed

The patient was a 49-year-old man admitted to our hospital on April 2021 due to dysuria and retention of a catheter for over a month because of urinary retention. His International Prostate symptom score (IPSS) was 23, and he was diagnosed with HIV infection in 2003, for which he was on regular ART (efavirenz 600 mg, zidovudine 300 mg, and lamivudine 150 mg). A digital rectal examination (DRE) revealed mild prostate hyperplasia without palpable nodules, and the serum PSA was 7.8 ng/ml. Preoperative MRI and CT scans (Fig. 1) indicated prostate and seminal vesicle enlargement and suggested a possible malignant tumor. The patient communicated that an external PET-CT examination showed no other tumors, however, no record of this information existed. Given the possibility of malignancy and the presence of dysuria, a transrectal puncture prostate biopsy was performed. The postoperative pathology was consistent with benign prostate hyperplasia (Fig. 2). The patient was negative for EBV infection, and the EBV serological status was negative for IgM and IgG. Accordingly, the diagnosis was challenging to determine. The possibility of malignancy in the seminal vesicle was still considered based on imaging examinations. Although a second biopsy was a higher risk, we considered repeating a puncture biopsy. However, the patient refused, because he wanted to regain urinary independence immediately. Additionally, he could not afford further treatment. After communicating with the patient and obtaining his consent, we eventually performed transabdominal radical resection of the seminal vesicle, the prostate, and the pelvic lymph nodes to establish the diagnosis, control tumor progression, and improve urination. Pathological specimens are shown in Fig. 3. The final diagnosis was PSBL associated with HIV (Fig. 4). EBV-related immunohistochemistry on puncture biopsy and surgical pathology suggested EBNA2(-) and EBER (-). Two weeks after the surgery, the urinary catheter was removed, and the patient regained normal urination ability with no apparent urinary incontinence. In July 2021, the patient was re-admitted due to urinary retention accompanied by fever, and was treated with cefminox as anti-infective therapy. Because of bilateral hydronephrosis, a left ureteral stent was placed, but the right ureteral stent procedure failed. A follow-up CT scan showed tumor progression (Fig. 5). The patient refused chemotherapy during this period and died on August 2021. Informed consent was obtained from the patient’s family members.

## Discussion

PSBL is a rare disease that can be challenging to differentiate from prostate cancer and other primary seminal vesicle tumors in clinical practice. Primary seminal vesicle carcinoma is relatively uncommon and is often invaded by carcinomas arising in nearby structures, such as the prostate, bladder, and rectum, which can complicate the differential diagnosis [6]. Early diagnosis of seminal vesicle carcinoma is often challenging due to the lack of immunohistochemical markers that distinguish it from invasive adenocarcinoma of adjacent organs [7].

Initially, we suspected prostate cancer in the 49-year-old male patient in this case, who was later diagnosed with BL. DRE suggested primary lymphoma in the prostate invading seminal vesicle. Other studies reported that DRE findings of prostate hyperplasia, hard consistency, and disappearance of the central fissure, or nodules in some patients are similar to primary prostate cancer [8, 9]. In our case, DRE suggested prostate hyperplasia, disappearance of the central fissure, and hard consistency without tenderness, raising concerns about prostate cancer. An overview of large patient samples showed that serum PSA was within 4 ng/ml [9]. The PSA test remains the only biomarker for detecting and monitoring prostate cancer [10]. Currently, PSA > 3 ng/ml, positive MRI prostate imaging shows the highest detection of prostate cancer [11]. These clinical features indicate prostate cancer, and a biopsy of the prostate was performed. However, we did not obtain the seminal vesicle pathology, which may be related to local anesthesia and insufficient understanding of the probability of primary seminal vesicle carcinoma. MRI/ultrasound combination can improve the positive rate of seminal vesicle invasion biopsy [12, 13]. The missed case emphasizes the need to improve biopsy accuracy. Cysto-prostato-seminal vesiculectomy with pelvic lymph node dissection should be adopted as the standard surgical approach for malignant tumors of seminal vesicle origin. However, as this condition is very rare, there is no standard treatment approach, and the outcomes are uncertain [14]. Partial resection of the seminal vesicles with postoperative adjuvant therapy has also been reported for the comprehensive treatment of primary tumors of the seminal vesicles [15].

The final diagnosis of BL has rarely been reported. In general, HIV-associated lymphomas are characterized by greater extranodal involvement than those found in patients who are negative for HIV. As in endemic and sporadic BL, the translocation of the MYC gene on chromosome 8 is present in almost 100% of BL cases associated with HIV. HIV-associated BL shares many histological features, such as positive CD20, CD10, and Ki-67 [2]. The histological features of the present case were similar to standard HIV-associated BL.

The commonly used treatment for BL is similar to that for other forms of lymphoma, and it mainly includes chemotherapy, radiotherapy, combined chemoradiotherapy, and resection of the tumor to relieve the local symptoms [1, 2]. Dunleavy and colleagues [16] reported that less toxic chemotherapy regimens (etoposide, doxorubicin, vincristine, cyclophosphamide, prednisone, and rituximab, DA-EPOCH-R), used at higher intensities than standard chemotherapy regimen of cyclophosphamide, doxorubicin, vincristine, and prednisone (CHOP), achieved higher response rates and overall survival in PLWH. The negative biopsy result reinforced our suspicion of prostate cancer, so we performed radical resection. It is generally recognized that the prognosis of BL is relatively poor [2]. Galicier [17] conducted a study on 63 PLWH with advanced disease who were treated with the LMB 86 regimen (escalated cyclophosphamide, doxorubicin, vincristine, and prednisolone and consolidation with cytarabine and etoposide), and found that the complete response rate was 70%, whereas 2-year overall survival was 47%. Rituximab has a vital role in BL treatment. Previous studies have shown that 10–30% dose adjustments may be necessary for CHOP-like chemotherapy regimens in patients with renal dysfunction and dialysis [18]. Inotuzumab ozogamicin and Brentuximab vedotin have potential as future treatment approaches. The emergence of immune checkpoint inhibitors may lead to better overall response rates, and using pembrolizumab in combination with R-CHOP therapy shows great potential. [19] Due to missed diagnosis, the present case progressed, missing the optimal treatment opportunity, and survived for less than four months.

The diagnostic and treatment process of this case was not ideal. Firstly, the preliminary diagnosis did not match the expected final pathology, which resulted in a lack of confidence in the clinical diagnosis and treatment process. Our clinical diagnostic and treatment strategies were insufficient, and the patient’s demands and economic ability significantly impacted the diagnostic and treatment processes. We need to learn from this experience and improve future diagnoses and treatments; Secondly, there were some mistakes in the diagnosis and treatment processes. HIV combined with BL in the seminal vesicle is rare condition, and there is a close relationship between seminal vesicle tumors and prostate tumors, which can be easily misdiagnosed. We hope this case can increase clinical physicians’ awareness and provide inspiration for diagnosing rare location tumors in the HIV population. Reported diagnostic and treatment strategies could benefit patients with similar conditions. This report will hopefully serve as cautionary advice to doctors, especially urologists, to change their approach and broaden the thinking model in the clinical diagnosis and treatment process.

## Conclusion

The diagnosis of primary seminal vesical Burkitt lymphoma is difficult and the prognosis is poorer than other lymphoma types. Earlier diagnosis and treatment may improve the survival of patients with Burkitt lymphoma.

## Data Availability

Derived data are available on reasonable request.

## Abbreviations

ART: Anti-retroviral therapy
BL: Burkitt lymphoma
CT: Computed tomography
DA-EPOCH-R: Etoposide, doxorubicin, vincristine, cyclophosphamide, prednisone, and rituximab
DRE: Digital rectal examination
MRI: Magnetic resonance imaging
PLWH: People living with HIV
PSA: Prostate-specific antigen
PSBL: Primary seminal vesicle Burkitt lymphoma
